# The use of automated sequential blood pressure in hypertension clinics compared with office and ambulatory blood pressure measurements

**DOI:** 10.1186/s43044-020-00087-9

**Published:** 2020-08-17

**Authors:** Kareem Mahmoud, Ayah ElAroussy, Yasser Baghdady, Wafaa ElAroussy, Heba ElDeeb

**Affiliations:** Cairo University, Cairo, Egypt

**Keywords:** Office blood pressure, Ambulatory blood pressure, Automated sequential blood pressure, White coat effect

## Abstract

**Background:**

Office blood pressure (OBP) measurement is the most common method of blood pressure measurement. However, it is associated with several pitfalls as white coat effect and masked hypertension. Ambulatory blood pressure monitoring (ABPM) is usually used for diagnosis of hypertension and elimination of white coat effect. This study aimed to assess the correlation and degree of agreement of the automated sequential blood pressure (ASqBP) with OBP and ABPM**.** Patients presented to hypertension clinic were included. Each patient had his blood pressure recorded by three methods: OBP using a digital sphygmomanometer device, unattended ASqBP using sequential BP devices with recording of the readings over 30 min, and ABPM that was performed within 48 h of office visit using portable BP devices with BP recording over 24 h.

**Results:**

We recruited 64 patients (age 50.0 ± 15.0 years and female gender 53.1%). We found a strong positive correlation between ASqBP and OBP readings (*r* 0.81 for SBP and 0.83 for DBP, *p* < 0.001). We also found a strong positive correlation between ASqBP and ABPM readings (*r* 0.74, *p* < 0.001). The ASqBP readings were lower than OBP (137.0 ± 16.8/86.4 ± 13.8 vs. 142.7 ± 15.5/88.5 ± 12.3) and close to ABPM readings (average 24 h, 134.0 ± 15.4/88.5 ± 12.3, and daytime, 135.8 ± 15.7/82.1 ± 13.7). For SBP readings, there was moderate agreement between ASqBP and AMBP (both average and daytime). For DBP readings, there was fair agreement between ASqBP and AMBP (both average and daytime).

**Conclusion:**

ASqBP measurement has good correlation with OBP and ABPM readings. Unattended automated office pressure has moderate degree of agreement with ABPM for the SBP& fair degree of agreement for the DBP. It can be used in the hypertension clinics to eliminate the problems of white coat effect and marked BP variability.

## Background

For decades, office blood pressure (OBP) has been used for diagnosis and follow-up of hypertension. However, the use of ambulatory blood pressure monitoring (ABPM) or home blood pressure monitoring (HBPM) for diagnosis of hypertension is now recommended [1]. This is due to several limitations observed with OBP such as white coat effect [2], inaccurate measurements [3], and lack of data on BP values during everyday activities [4].

Automated sequential blood pressure (ASqBP) then came to action with its fully automated electronic sphygmomanometer that helps to record multiple BP readings without the need for physician while the patient is resting in a quiet place [5]. In 2011, the Canadian Hypertension Education Program (CHEP) validate ASqBP as an alternative to manual office BP [6]. This made European hypertension guidelines considered the use of ASqBP, when feasible, to improve BP measurements reproducibility and get an office BP values closer to the daytime ABPM and HBPM [1]. Despite OBP was the cornerstone of many previous clinical trials, the Systolic Blood Pressure Intervention Trial (SPRINT) [7] used unattended office BP measurement instead. After the positive result of SPRINT trial, a controversy was raised about the relationship between OBP and ASqBP measurements as well as the use of ASqBP in daily clinical practice.

Our study aimed to compare ASqBP measurement to AMBP measurement and OBP measurement in the diagnosis and monitoring of hypertensive patients in the Egyptian hypertension clinic.

## Methods

### Subjects

This was a cross-sectional observational study that was conducted from January 2017 to May 2017. We included patients who attended the Hypertension Specialized Clinics—settled by the Egyptian Society of Hypertension—at Cairo University Hospital, to check their medical status, and/or control their blood pressure. Written informed consent was obtained from all subjects enrolled in the study. The study was approved by the local ethics committee. We excluded patient who refused to sign informed consent and failed to perform or denied the use of ASqBP.

### Methods

All patients were subjected to:

- Medical history including age; gender; cardiovascular risk factors, e.g., smoking, dyslipidemia, and diabetes mellitus; medical illness, e.g., chronic kidney disease (CKD); and current antihypertensive medications.
- Body mass index (BMI) and waist circumference (WC).
- Blood pressure measurements.

Systolic and diastolic blood pressure readings were obtained through the following methods:

1. *Office BP (OBP)* measurement according to the European Society Guidelines for the Management of Hypertension [1] using a digital sphygmomanometer device for blood pressure measurement (Omron-5 automated device).
2. *Automated Sequential BP measurements (ASqBP)*: The Mobil-O-Graph ®, PWA, was used to measure the unattended sequential blood pressure over a period of half an hour in a quiet room. The measurements were taken every 2 min, and the results were averaged, after excluding the first and final ones [5].
3. *Ambulatory BP measurements (ABPM)*: ABPM was performed with the patient wearing a portable BP measuring device, usually on the non-dominant arm using (Holter system, Model: DMS 300-4A, USA) every half an hour in daytime and every hour during the night according to patient sleep and awake time for a 24-h period. The test was done within 48 h of clinic visit. For each patient, a cuff containing an inflatable bladder of correct length and width appropriate for him is used. The measurements were downloaded to a computer and at least 70% of the readings during daytime and night-time periods should be satisfactory, or else the monitoring should be repeated [8].

- Labs including lipid profile, urea, and creatinine; urine analysis for microalbuminuria; and blood sugar measurement (random, fasting, and HbA1c).

### Objectives

- To assess the correlation and degree of agreement of the automated sequential blood pressure (ASqBP) with OBP and ABPM**.**

### Statistical methods

Statistical analysis was performed using S-Plus Statistical Software (SPSS) for Windows (version 17.0, SPSS Inc. Chicago, Illinois). Continuous variables were presented as mean ± standard deviation. Categorical variables were presented as numbers and percentages. Continuous variables were compared using Student’s *t* test while categorical variables were compared using chi-square and Fischer’s exact tests. Degree of agreement between sequential and ambulatory blood pressure measurements was evaluated using kappa test and interpreted according to the result into none to slight (0.01–0.20), fair (0.21–0.40), moderate (0.41–0.60), substantial (0.61–0.80), or perfect (0.81–1.00) [9]. Correlation between continuous variables was done using the Pearson correlation. A *p* value of less than 0.05 was considered significant.

## Results

Sixty-four patients were included in this study. Table 1 shows baseline characteristics of our studied patients. They were mostly middle-aged obese females. Their office BP readings were mildly elevated especially SBP values. One fifth of the group were smokers. The most common risk factors in our patients were diabetes mellitus (25%) and dyslipidemia (17.2%). Regarding antihypertensive medications, beta-blockers were the most commonly used drugs (45.3%) followed by calcium-channel blockers (25%). The combination drugs were used in about quarter of the patients—mainly ACEI or ARB plus thiazide diuretics.

**Table 1:** Baseline characteristics of the studied population, means ± SD or *N* (%)

Characteristic	Value
Age (years)	50.0 ± 5.0
Female	34 (53.1)
BMI (kg/m^2^)	31.3 ± 6.3
WC (cm)	95.0 ± 12.4
Office HR (bpm)	76.4 ± 12.0
Office SBP (mmHg)	142.7 ± 15.5
Office DBP (mmHg)	88.5 ± 12.3
Diabetes Mellitus	16 (25.0)
Smoking	13 (20.3)
Dyslipidemia	11 (17.2)
COPD	9 (14.1)
CKD	5 (7.8)
BPH	1 (1.6)
Depression	5 (7.8)
CAD	7 (10.9)
Heart failure	2 (3.1)
Stroke	1 (1.6)
Type of antihypertensive medications	
Beta-Blocker	29 (45.3)
CCB	16 (25.0)
ACEI	11 (17.2)
ARB	1 (1.6)
Diuretics	6 (9.6)
Centrally acting drugs	1 (1.6)
Alpha-Blocker	2 (3.1)
Spironolactone	2 (3.1)
Combinations	15 (23.4)

*ACEI* angiotensin-converting enzyme inhibitor, *ARB* angiotensin receptor blocker, *BMI* body mass index, *BPH* benign prostatic hyperplasia, *CAD* coronary artery disease, *CCB* calcium channel blocker, *CKD* chronic kidney disease, *COPD* chronic obstructive pulmonary disease, *DBP* diastolic blood pressure, *HR* heart rate, *SBP* systolic Blood pressure, *WC* waist circumference

**Fig. 1:**
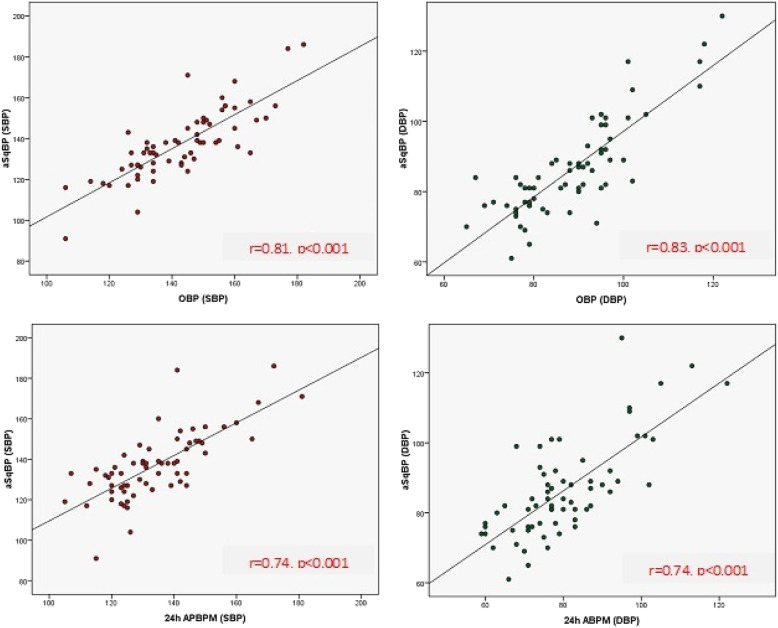
Correlations between OBP and ASqBP as well as 24 h ABPM and ASqBP

Table 2 shows the BP readings as measured with different methods of BP measurements (e.g., office, ambulatory, and automated sequential BP measurements). There was strong positive correlation between ASqBP and OBP readings (for both SBP and DBP values). We also found a strong positive correlation between ASqBP and AMBP readings (for both SBP and DBP values) (Fig. 1).

**Table 2:** Comparison and correlations between OBP, ABPM (average 24 h and daytime), and ASqBP measurements, means ± SD

	OBP	ABPM (average 24 h)	*R*	*P* value
SBP (mmHg)	142.7 ± 15.5	134.0 ± 15.4	0.64	> 0.001
DBP (mmHg)	88.5 ± 12.3	80.2 ± 13.2	0.66	< 0.001
	**OBP**	**ABPM (daytime)**	***R***	***P*** **value**
SBP (mmHg)	142.7 ± 15.5	135.8 ± 15.7	0.64	> 0.001
DBP (mmHg)	88.5 ± 12.3	82.1 ± 13.7	0.66	< 0.001
	**OBP**	**ASqBP**	***R***	***P*** **value**
SBP (mmHg)	142.7 ± 15.5	137.0 ± 16.8	0.81	> 0.001
DBP (mmHg)	88.5 ± 12.3	86.4 ± 13.8	0.83	> 0.001
	**ABPM (average24 h)**	**ASqBP**	***R***	***P*** **value**
SBP (mmHg)	134.0 ± 15.4	137.0 ± 16.8	0.74	> 0.001
DBP (mmHg)	88.5 ± 12.3	86.4 ± 13.8	0.74	> 0.001
	**ABPM (daytime)**	**ASqBP**	***R***	***P*** **value**
SBP (mmHg)	135.8 ± 15.7	137.0 ± 16.8	0.74	> 0.001
DBP (mmHg)	82.1 ± 13.7	86.4 ± 13.8	0.73	> 0.001

*ABPM* ambulatory blood pressure measurement, *ASqBP* automated sequential blood pressure, *DBP* diastolic blood pressure, *OBP* office blood pressure, *SBP* systolic blood pressure

For SBP readings, there was moderate agreement between ASqBP and AMBP (both average and daytime). For DBP readings, there was fair agreement between ASqBP and AMBP (both average and daytime) (Table 3).

**Table 3:** The degree of agreement between ASqBP and ABPM

	Kappa	*P* value
ASqBP (systolic):		
• 24 h ABPM • Daytime ABPM	0.48 0.51	< 0.001 < 0.015
ASqBP (diastolic):		
• 24 h ABPM • Daytime ABPM	0.30 0.38	< 0.001 < 0.003

## Discussion

Our study shows that ASqBP showed good correlation with AMBP and OBP when it is used for BP measurement in the Egyptian Hypertension Clinics. There was moderate degree of agreement between ASqBP and ambulatory SBP (24 h and daytime) and fair degree of agreement between ASqBP and ambulatory DBP (24 h and daytime).

Hypertension is very common medical problem affecting 26.3% of adult Egyptians with only 38% of them were aware of having high blood pressure. It was found that only 24% of hypertensive patients were receiving the antihypertensive medications, with control rates (i.e., < 140/90 mmHg) were 8% [10].

Office BP measurement is the routine in clinical evaluation of patients and follow-up. Because of the white coat effect, several patients have been labeled hypertensive and were prescribed anti-hypertensive medications for life, with subsequent hypotensive episodes. The use of ambulatory BP monitoring has solved to a great extent this problem of labile hypertension, and/or white coat effect. However, it is troublesome as usually disturbed by the effect of inflating cuffs. Therefore, the measurements might not reflect the basal conditions.

Theoretically, ASqBP eliminates the human error as well as attenuates the white coat effect, since it allows for multiple readings to be taken in unattended fashion. The Canadian guidelines recommended the use of automated devices as the method of choice for office BP measurement [11]. In the SPRINT study, BP was measured using an automated BP device (Omron HEM 904), which was preset to wait 5 min before measurements and to take average of three measurements, with a 1-min interval, while sitting in a quiet room unobserved [7]. This emphasizes the clinical importance of using the automated devices for accurate BP measurements in clinical trials.

In the current study, we aimed at comparing sequential blood pressure measurement with both OBP and ambulatory monitoring, to correlate between their readings, and whether automated sequential BP can eliminate the pitfalls of blood pressure measurement as white coat effect.

Our results showed that ASqBP measurement is significantly lower than the OBP measurement. Scherpbier-de Haan et al. revealed in their study, on 83 adult patients, that 30-min ASqBP measurements better reflects the patient’s true BP than standardized OBP does. Their mean 30-min ASqBP readings were 7.6/2.5 mmHg (95% confidence interval [CI] = 6.1 to 9.1/1.5 to 3.4 mmHg) lower than OBP readings [12].

Leenen et al. [13] used ASqBP in a community BP survey. ASqBP was seen to have several advantages over manual BP including more accurate and consistent readings without the need for extensive training of research staff. The Ontario Survey on the Prevalence of High Blood Pressure (ON-BP) recorded ASqBP using the BpTRU in 2551 adult subjects, with BP readings also being performed using a standard mercury sphygmomanometer in a sample (*n* = 238) of this population [14]. ASqBP readings were slightly lower (115/71 mm Hg) than the mean manual BP (118/74 mm Hg). Subsequently, Wilkins et al. [15] reproduced these findings in a national Canadian health survey, using the BpTRU to assess BP status. Bos and Bui showed a similar result with ASqBP readings which were considerably lower than the readings of the OBP. The mean systolic ASqBP was 22.8 mmHg lower than the mean systolic OBP (95% CI, 19.8–26.1 mmHg). The mean diastolic ASqBP was 11.6 mmHg lower than the mean diastolic OBP (95% CI, 10.2–13.1 mmHg). Considerable differences between OBP and ASqBP existed in patients with and without suspected white-coat hypertension, and differences were larger in individuals aged 70 years or older. These results come in agreement with the findings of the current study, where the ASqBP measurements were lower than OBP.

Beckett and Godwin compared BpTRU automatic blood pressure monitor to mean daytime 24-h ambulatory blood pressure monitoring in the assessment of BP in 481 patients with hypertension. The group mean of the average of five BpTRU readings was not statistically different from the 24-h daytime mean on ABPM with mean ± SD of 140.0 ± 17.71/79.8 ± 10.46 vs 141.5 ± 13.25/79.7 ± 7.79 mmHg, respectively. Within patients, BpTRU average correlated significantly better with daytime ambulatory pressure than did clinic averages (*r* = 0.571 and *r* = 0.145, respectively) [16]. These results are different from the values of the current study, where the readings of the ASqBP monitoring were statistically higher than the daytime mean ± SD ABPM measurements’ values which were 137.0 ± 16.8 SBP, vs 135.8 ± 15.7 mmHg, and 86.4 ± 13.8 DBP vs 82.1 ± 13.7 mmHg, (*p* < 0.001 for both). However, there was good correlation between ASqBP (both systolic and diastolic) and daytime ABPM measurements (*r* = 0.74, and 0.73, *P* < 0.0001 respectively).

Godwin et al. studied the manual and automated office measurements in relation to awake ambulatory blood pressure monitoring by taking single automated sequential BP measurement and the mean of three OBP on different sets for 654 hypertensive patients; their results showed that the single ASqBP correlates better than the three mean OBP with the daytime ABPM which is similar to the results of the present study. In this study, Pearson correlations were as following: daytime ABPM vs ASqBP systolic/diastolic (*r* = 0.591 and 0.587 respectively) and for daytime ABPM vs mean OBP systolic/diastolic (*r* = 0.173 and 0.306 respectively) [17].

To the best of our knowledge, this is the first study conducted on Egyptian hypertensive patients using the unattended ASqBP device which revealed good correlations with AMBP and emphasized that we should not only depend on OBP readings for diagnosis and follow-up medications. Meanwhile, ASqBP might be beneficial in two aspects. First, it may be cost effective by decreasing the need of ambulatory blood pressure reducing the cost of its use and decreasing the number of visits to outpatient clinics. Second, it can help to reduce physician patient contact during office visits in the current era of COVID-19.

Temporal timing of BP measurements is considered a limitation of our study. During office visit, we recorded OBP and ASqBP readings, while AMBP recording was done either on the same day or within 48 h from the office measurement, which could bias BP readings. Another limitation is the small number of the patient in the study.

## Conclusions

We conclude that ASqBP has good correlation with AMBP. For SBP readings, there was moderate agreement between ASqBP and AMBP (both average and daytime). For DBP readings, there was fair agreement between ASqBP and AMBP (both average and daytime). Unattended sequential BP measurement could overcome the problem of white coat effect that is frequently encountered with office blood pressure measurement.

## Data Availability

The dataset supporting the results and conclusions of this article will be available from the corresponding author on request.

## Abbreviations

ABPM: Ambulatory blood pressure monitoring
ACEI: Angiotensin converting enzyme inhibitor
ARB: Angiotensin receptor blocker
ASqBP: Automated sequential blood pressure
BMI: Body mass index
BP: Blood pressure
BPH: Benign prostatic hyperplasia
CAD: Coronary artery disease
CCB: Calcium channel blocker
CKD: Chronic kidney disease
COPD: Chronic obstructive pulmonary disease
DBP: Diastolic blood pressure
HBPM: Home blood pressure monitoring
HR: Heart rate
OBP: Office blood pressure
SBP: Systolic blood pressure
WC: Waist circumference
